# Leptin Influence in Craving and Relapse of Alcoholics and Smokers

**DOI:** 10.4021/jocmr1159w

**Published:** 2013-04-23

**Authors:** Aline S. Aguiar-Nemer, Mayla C. F. Toffolo, Claudio Jeronimo da Silva, Ronaldo Laranjeira, Vilma A. Silva-Fonseca

**Affiliations:** a Departamento de Nutricao, Instituto de Ciencias Biologicas, Universidade Federal de Juiz de Fora, Juiz de Fora, Minas Gerais, Brasil; b Unidade de Pesquisas em Alcool e Drogas (UNIAD) - Departamento de Psiquiatria - UNIFESP, Sao Paulo, Brasil; c Nucleo de Ciencias Comportamentais e do Desenvolvimento, Departamento de Fisiologia e Farmacologia, Instituto Biomedico, Universidade Federal Fluminense, Rio de Janeiro, Brasil

**Keywords:** Leptin, Alcoholism, Smoking, Drug dependence, Review

## Abstract

Leptin inhibits signaling of dopamine in the nucleus accumbens, suggesting its role in regulating stress and its possible involvement in the neurobiology of reward system. The aim of this study was to review of the literature on the influence of leptin in the craving for alcohol and tobacco and whether there is already evidence that leptin may be a biomarker to indicate risk for craving and relapse. The review used as data bases Medline, LILACS and SciElo in the period between 2000 and 2012. Keywords were leptin, substance use disorders, craving and withdrawal, in Portuguese and English. Only 12 articles were met the inclusion criteria, relating leptin with craving in alcoholics (n = 10) and smokers (n = 2). No studies were found in the LILACS database. Leptin levels increase during abstinence and this may be related to a reduction of dopaminergic action in mesolimbic system, resulting in a greater intensity of craving and maintenance of addictive behavior. Although there are few studies, the most recent results indicate the usefulness of leptin as a marker of risk for relapse among smokers and alcoholics in abstinence.

## Introduction

Leptin is a polypeptide hormone secreted mainly by adipose tissue. It acts on the central nervous system by inhibiting food intake and increasing energy expenditure, with an important role in controlling body weight [1].

The presence of an intense desire and urgency to the drug, known as craving for the substance, is important for the diagnosis of chemical dependency [2]. Although this criterion is important for the detection of high risk for relapse, there are no measures or biological markers that could objectively signalize the proximity of this risk. In this context, studies have been conducted to identify the influence of adipokines, particularly leptin, in neurobiology of addiction in an attempt to identify a possible marker for the subjective sense of compulsion to use psychoactive substances.

In 2001, it was published the first evidence that the pathway involved in the neuroendocrine energy expenditure, regulation of appetite and craving for food, can also interfere with the pathophysiological mechanism of addiction. The study demonstrated the involvement of leptin in the mechanisms of craving and confirmed its action in the hypothalamus and dopaminergic neurons. This initial study indicated that increased plasma concentration of leptin was associated with increased craving for alcohol in alcoholics treated for detoxification [3].

However, few studies investigated the mechanisms involved and the relationship between plasma changes in the concentration of leptin and its influence on reward, relapse and maintenance of abstinence. Besides, the studies were focused on alcohol, and only a few explored whether there is effect of leptin in the craving and relapse of other drugs, for example, tobacco.

In this context, this review aims to describe what it is in the literature about the influence of leptin in the craving for alcohol and tobacco and whether there is already evidence that leptin may be a biomarker to indicate risk for craving and relapse.

## Literature Study

Searches were performed in Medline (National Library of Medicine), SciElo and LILACS (Sistema Latino-Americano e do Caribe de Informacao em Ciencias da Saude) databases, considering articles published in English, Portuguese and Spanish language between 2000 and 2012. The review was conducted from March to April 2012. The selection of articles was based on studies on humans and related to the objective of the review. The following key words were used: leptin, craving, substance use disorders and withdrawal. There were excluded studies that related, among addicts, leptin levels with nutritional status, body fat and body mass index (BMI), without mentioning the possible influence on abstinence and craving. In addition to the search on databases, a manual search in reference lists of selected articles was also performed. Review articles and other references important for definitions were also included.

## Results

Forty articles in the Medline database and one article in the SciElo database, published in English, were selected. One article published in German and another twenty-nine studies that they did not relate leptin and/or craving in their title, abstract or text were excluded. Thus, this review was performed with the analysis of 12 articles relating leptin with craving in alcoholics (n = 10) and smokers (n = 2). No studies were found in the LILACS database.

## Leptin on craving in alcoholics

Kiefer et al [3, 4] initiated studies to assess whether the craving for alcohol can be modulated by leptin. The authors were motivated by the fact that leptin regulates the hypothalamic-pituitary-adrenocortical (HPA) hormone axis (HPA), that the feedback endocrine system is altered in alcoholics and a strong apparent similarity between the reward of food and drugs.

In two independent studies, one with 20 individuals [3] and the other extending the sample to 78 alcoholics [4], men and women, the authors demonstrated that serum leptin levels were higher in the 14th day after alcohol withdrawal compared with nonalcoholic individuals (controls). However, there was significant reduction in plasma leptin levels between the 1st and 14th days of abstinence. Even so, the values ??in this period were higher than those shown by control subjects. Alcohol craving decreases from the first to the fourteenth day of abstinence. Additionally, the authors showed a positive association between leptin levels and the measurement of craving during this short period of abstinence [3, 4].

In order to understand the possible pathophysiological mechanism explaining alcohol consumption and increased plasma leptin levels in eutrophic alcoholics Kiefer et al [5] conducted a study, with male alcoholics in detoxification, evaluating the possible influence of tumor necrosis factor - alpha (TNF-α) in this mechanism, since the adipose tissue synthesizes cytokines and there is evidence of increased leptin levels after injection of TNF-α. There was a positive correlation between plasma levels of leptin and TNF-α, which is also related to the duration of alcohol consumption. The authors suggested the existence of a possible vicious cycle: alcohol intake, increase of TNF-α, enhanced leptin secretion, enhanced alcohol craving and, consecutively, increased alcohol intake.

Wrust et al [6] found no significant difference between serum leptin and leptin levels in alcoholic patients between the first and seventh days of abstinence. According to these authors, leptin was not identified as a marker of alcohol intoxication, contradicting the results of the studies cited above.

However, Kraus et al [7] evaluated alcoholics with 10 days of abstinence, suggesting that serum leptin increases in abstinence and correlates with the scale of craving only among women. In men, leptin levels showed no significant results with the scale of assessment of craving. It was the first study that examined men and women separately.

Kiefer et al [8] studied the leptin levels during 12 weeks of withdrawal in patients treated with naltrexone; acamprosate; naltrexone plus acamprosate or placebo. There was an increase in leptin levels between the placebo groups. However, treatment with the drugs combined promoted a significant reduction of plasma leptin. The change in leptin over four weeks was positively correlated with self-assessment of alcohol craving. These results suggest that increased leptin during withdrawal precedes the resumption by alcohol consumption. The decreased leptin during the drug treatment was directly associated with increased time of withdrawal.

Hillemacher et al [9] also presented results to strengthen the association between leptin levels and alcohol craving in both men and women. However, the authors found no difference between sexes, as suggested previously [7].

Recently, Toffolo et al [10] observed higher serum leptin levels, leptin/BMI and leptin/% body fat among women recently abstinent (abstinent 1 - 3 months), even after adjustment for BMI and % BF. Leptin levels between men and women abstinent for longer (abstinent for 4 months or more) were close to the levels of men and women not abstinent. The authors suggested that the increase in leptin in the beginning of withdrawal may be a factor which influences the drug cravings.

## Leptin on craving in smokers

There are only two studies evaluating the craving for tobacco and changes in leptin levels. These were published recently and conducted due to evidence found among alcoholics. In a study by Van der Goltz et al [11], there was a positive association between the intensity of nicotine craving and plasma leptin, suggesting that leptin regulates the dopaminergic transmission during withdrawal and therefore modulate the craving for this drug. The results found by al’Absi et al [12] showed a significant positive association between leptin concentration and craving for cigarettes after 24 hours of abstinence.

## Discussion

Leptin acts in the regulation of the HPA and its action changes the gene expression of corticotropin-releasing hormone (CRH) and pro-opiomelanocortin (POMC) in the hypothalamus. In addition, it binds to specific receptors located on dopaminergic neurons in the ventral tegmental area (VTA), inhibiting the signaling of dopamine in the nucleus accumbens [13]. These actions suggest its important role in regulating stress and its possible involvement in the neurobiology of reward system of the drug [14].

However, the mechanisms underlying the association between plasma leptin and craving are still unknown and there is few literature on the subject. Most of the studies analyzed present the results without separating men and women alcoholics [3, 4, 6, 8] and smokers. [11, 12]. Only three studies assessed men and women separately [7, 9, 10]. Testosterone inhibits the secretion of leptin by adipocytes [15]. For this reason, sexual difference may be considered, since women leptin levels are three times greater than men, even with the same BMI [16].

Although one study indicated a positive correlation between leptin and craving among women with 10 days of abstinence [7] and others indicate that there are higher levels of leptin among women recently abstinent (1 - 3 months) compared to women with more 4 months of abstinence [10], they do not make clear whether there is greater intensity of craving between them, but it suggests that leptin may influence relapse during this critical period of abstinence.

Another relevant factor is the small number of participants included in some studies (ranging from 20 to 86 participants). This limitation was taken up and discussed by the authors [3-6, 10-12]. However, studies with more than 100 individuals, conducted in 2004 [7], 2005 [8] and 2007 [9], concluded that the increase in plasma leptin concentration precedes relapse suggesting that it could be a biological marker of relapse among women [7].

Leptin and its associated to neural circuits provide an intriguing field for future investigations in the field of pharmacological treatment of addiction [17].

### Conclusion

The increase in leptin levels during withdrawal can promote the reduction of the dopaminergic action in mesolimbic system, resulting in a greater intensity of craving and maintenance of addictive behavior. In addition to leptin, the specificities of each psychotropic drug, the individual characteristics of dependents, should be considered as it may influence the neurobiology of craving and relapse.

Although there are few studies, the most recent results indicate the usefulness of leptin as a marker of risk for relapse among smokers and abstinent alcoholics. Future research is still needed to demonstrate the neuroendocrine mechanism of dependence. It might be interesting for the development of drug strategies to consider leptin in the therapy of tobacco dependence and alcohol.
